# 

**Published:** 1895-09

**Authors:** 


### CONTENTS.

ORIGINAL COMMUNICATIONS—
- THE MANAGEMENT OF LABOR IN CASES OF MINOR PELVIC DEFORMITY. By Joseph Eve Allen, M.D., Augusta, Ga. .... 385
- ON PNEUMOTHORAX. By Charles Lewis Allen, M.D., New York .... 392
- A CASE OF TETANUS AFFECTING THE MUSCLES OF JAWS ONLY. By N. C. Mitra, M.A., M.B., Ranchi, Bengal, India .... 400
- THE PHYSIOLOGY OF THE BILE AND GALL-BLADDER, AND THE ETIOLOGY OF CHOLELITHIASIS. By H. H. Levy, M.D., Richmond, Va. .... 402
- WHAT SHALL BE DONE WITH THE HOMICIDAL CRANK. By Theodore Diller, M.D., Pittsburg, Pa. .... 405

SOCIETY REPORTS—
- ALLEGHENY COUNTY MEDICAL SOCIETY .... 411

CORRESPONDENCE—
- THE CHEMICAL ACTION OF STRYCHNINE, QUININE, ACONITINE, AND ATROPINE .... 419

EDITORIAL—
- BICYCLING FOR WOMEN .... 421
- THE COTTON STATES AND INTERNATIONAL EXPOSITION .... 423
- INSANITY, EXPERT MEDICAL TESTIMONY AND THE COURTS .... 425

SELECTIONS AND ABSTRACTS—
- EYE, EAR, NOSE, AND THROAT .... 427
- PRACTICAL CHEMISTRY AND PHARMACY .... 432
- OBSTETRICS AND GYNECOLOGY .... 436
- SURGERY .... 438

MEDICAL ITEMS .... 443
BOOK REVIEWS .... 445
ADVERTISERS' INDEX TO ADVERTISEMENTS .... xii
MEDICAL ITEMS—CONTINUED .... x, xi
CLASSIFIED INDEX TO ADVERTISEMENTS .... xxxv

